# Single-course antenatal corticosteroids is related to faster growth in very-low-birth-weight infant

**DOI:** 10.1186/s12884-020-03510-w

**Published:** 2021-01-12

**Authors:** Jiajia Jing, Yiheng Dai, Yanqi Li, Ping Zhou, Xiaodong Li, Jiaping Mei, Chunyi Zhang, Per Trop Sangild, Zhaoxie Tang, Suhua Xu, Yanbin Su, Xiaoying He, Yanna Zhu

**Affiliations:** 1 Department of Maternal and Child Health, School of Public Health, Sun Yat-sen University, No. 74 Zhongshang 2nd Road, Guangzhou, 510080 China; 2 Department of Neonatology, Foshan Woman and Children’s Hospital, Foshan, China; 3 Comparative Pediatrics and Nutrition, University of Copenhagen, Copenhagen, Denmark; 4 Department of Neonatology, Shenzhen Bao’an Maternal and Child Health Hospital, Shenzhen, China; 5 Department of Neonatology, Shenzhen Nanshan People’s Hospital, Shenzhen, China; 6 Department of Neonatology, Shenzhen Maternity & Child Health Care Hospital, Shenzhen, China; 7 Department of Neonatology, Guangdong Women and Children Hospital, Guangzhou, China; 8 Department of Pediatrics and Adolescent Medicine, Rigshospitalet, Copenhagen, Denmark

**Keywords:** Antenatal corticosteroids, Growth, Nutrition, Very low birth weight

## Abstract

**Background:**

Antenatal corticosteroids (ACS) treatment is critical to support survival and lung maturation in preterm infants, however, its effect on feeding and growth is unclear. Prior preterm delivery, it remains uncertain whether ACS treatment should be continued if possible (repeated course ACS), until a certain gestational age is reached. We hypothesized that the association of single-course ACS with feeding competence and postnatal growth outcomes might be different from that of repeated course ACS in very-low-birth-weight preterm infants.

**Methods:**

A multicenter retrospective cohort study was conducted in very-low-birth-weight preterm infants born at 23–37 weeks’ gestation in South China from 2011 to 2014. Data on growth, nutritional and clinical outcomes were collected. Repeated course ACS was defined in this study as two or more courses ACS (more than single-course). Infants were stratified by gestational age (GA), including GA < 28 weeks, 28 weeks ≤ GA < 32 weeks and 32 weeks ≤ GA < 37 weeks. Multiple linear regression and multilevel model were applied to analyze the association of ACS with feeding and growth outcomes.

**Results:**

A total of 841 infants were recruited. The results, just in very-low-birth-weight preterm infants born at 28–32 weeks’ gestation, showed both single and repeated course of ACS regimens had shorter intubated ventilation time compared to non-ACS regimen. Single-course ACS promoted the earlier application of amino acid and enteral nutrition, and higher rate of weight increase (15.71; 95%CI 5.54–25.88) than non-ACS after adjusting for potential confounding factors. No associations of repeated course ACS with feeding, mean weight and weight increase rate were observed.

**Conclusions:**

Single-course ACS was positively related to feeding and growth outcomes in very-low-birth-weight preterm infants born at 28–32 weeks’ gestation. However, the similar phenomenon was not observed in the repeated course of ACS regimen.

## Background

Antenatal corticosteroids (ACS) treatment is commonly used worldwide as the standard treatment for pregnant women at risk of preterm labor [1]. European consensus guidelines for the management of neonate respiratory distress syndrome (RDS) in preterm infants recommended that single-course ACS should be used at least 24 h before delivery < 34 gestational age (GA). Repeated course ACS may be used if preterm labor is estimated to be < 32 GA and the first course was given more than 1–2 weeks earlier [2]. Different ACS regimens were critical to support survival [3] and lung maturation [4, 5], whereas, had unclear effects on feeding and growth in very-low-birth-weight (VLBW) preterm infants. Moreover, the risk-to-benefit ratio for repeated course ACS application was still ambiguous [1, 6, 7].

The effects of ACS were different between single and repeated course treatments. Meta-analyses [8, 9] showed that single-course ACS can effectively reduce the risk of RDS, necrotizing enterocolitis (NEC), and infection. Repeated course ACS can also decrease the risk of RDS, but accompanied some side effects. For instance, several studies reported that repeated course ACS seemed to be negatively correlated with weight growth in the uterus [10] or not [11, 12]. Whereas, the similar reduction trend of weight was not observed when at discharge [13] or in 2–3 years later [14–17]. We should notify that those results adjusted for the maternal factors but ignored postnatal factors, such as nutritional factors. Many studies have demonstrated that ACS improved intestinal maturation in a manner similar to lungs [18–20]. However, there was a lack of evidence on the relationship between ACS and nutritional outcomes. This has prompted us to conduct further studies on the association of ACS with postnatal growth and feeding outcomes.

In summary, we aimed to explored whether ACS, with single-course or repeated course were associated with growth, nutritional outcomes (e.g. time to initiate parenteral and enteral feeding, time to reach full enteral feeding) and clinical outcomes (e.g. days on mechanical ventilation, NEC incidence, mortality) in VLBW infants based on a retrospective cohort.

## Materials and methods

### Study design and subjects

The retrospective cohort study was a part of the NEOMUNE study [21], which was performed in five neonatal intensive care units (NICUs) from 2011 to 2014 in Guangdong province, China. Inclusion criteria were infants inborn or transferred to the NICU within 24 h after birth, < 37 weeks’ gestation and < 1500 g at birth, survived for at least 24 h. Exclusion criteria included major congenital abnormalities or congenital metabolic diseases.

### Exposure and outcomes

The VLBW preterm infants were classified as non- ACS group, single-course ACS group and repeated course ACS group. Since a systematic review reported that the scope of GA for which the ACS provides benefits has been controversial [14]. In the present study, the participants were stratified by gestational age as three groups-GA < 28 weeks, 28 weeks ≤ GA < 32 weeks and 32 weeks ≤ GA < 37 weeks. Single-course ACS in the study meant that 24 mg of dexamethasone intramuscularly in 6 mg per dose every 12 h for a total of four doses. Repeated course ACS was defined in this study as two or more courses ACS (more than single-course). The pregnant women exposed to fewer than 4 doses were assigned to the single-course ACS group. Information including maternal and neonatal demographics (maternal age, delivery mode, GA, biological sex, APGAR score at 5 min and anthropometrics at birth), ACS use, type of enteral nutrition (complete breastfeeding, mix feeding, 100% preterm formula milk), nutritional and clinical data (initiation day of parenteral and enteral feeding, time to reach full enteral feeding, days on intubated ventilatory and non-invasive ventilator, NEC, intrauterine growth retardation [IUGR] and so on) were collected. In the present study, complete breastfeeding meant 100% own mother’s milk. Postnatal weight data were meticulously measured weekly during hospitalization. Time to reach full enteral feeding was defined as time to achieving enteral feeding volumes of 150 mL·kg^− 1^·d^− 1^ [22]. IUGR was defined as retardation of fetal development resulting in small size in relation to gestational age, using less than the tenth percentile as a cutoff growth criterion [23]. The recommended formula for growth velocity was the exponential method published by Patel et al. [24]. Growth velocity (g·kg^− 1^·d ^− 1^) = [1000 × ln (Weight Day _n_/Weight Day _1_)] / (Day _n_ – Day _1_). All data were collected until 37 postmenstrual age or at discharge (including discharge upon parental request), or death.

### Statistical analyze

All statistical analyses were performed using the software SPSS 25.0 (SPSS Inc. Chicago, IL). Cases with lost (or missed) ACS information were eliminated. Data were summarized using means and SDs, medians and interquartile ranges (IQRs), numbers and percentages, as appropriate. Kruskal-Wallis *H* test and *χ*^2^ test were used to compare the nutritional and clinical outcomes among ACS groups. Mann–Whitney test was used for post hoc analysis. Multiple linear regression was used to analyze the association of ACS with nutritional and clinical outcomes. Multilevel model was used to analyze the association between ACS and early weight growth trajectories. In multilevel model, the coefficients for ACS were related to differences in mean weight, for interactions between the ACS and time could be interpreted to indicate differences in mean weight varied over time (weight increase rate).

### Missing data

Data in this study were collected from routine records of clinical systems, for this reason, the proportions of missing data in each variable were small, including 0.95% for maternal age, 0.59% for multiple births, 0.23% for cesarean section, 3.68% for birth length, 0.95% for APGAR score, 0.83% for intubation ventilation, 1.55% for non-invasive ventilation, 2.85% for extra oxygen supply, 3.92% for regain birthweight, 0.24% for inpatient time, and 0.59% for amino acid introduction and duration. Multiple imputation was not performed.

### Sensitivity analyses

We used stratified analysis to address confounding factor GA, double entry to reduce information bias and missing data bias. Additionally, the inclusion and exclusion criteria were strictly followed to avoid selection bias. The intrauterine status of some unmeasured confounding may have an impact on nutrition and growth outcomes. In order to ascertain the sensitivity of our results to different health status in utero, analyses were performed to evaluate the association of ACS regimens with nutritional outcomes and postnatal growth in IUGR or non-IUGR VLBW preterm infants in supplementary material.

## Results

A total of 1178 VLBW preterm infants were identified. Two hundred forty-six cases were excluded for major congenital abnormalities, metabolic diseases or transfer to another hospital within 24 h of birth. Ninety-one cases without ACS information were eliminated. As a result, 841 VLBW preterm infants were recruited in the study, including 456 (54.2%) in non-ACS group, 162 (19.3%) in single-course group and 223 (26.5%) in repeated course group. Average follow-up time was 39 days. The median GA and birthweight were 30.1 (28.9–31.6) weeks and 1300 (1150–1400) g, respectively.

### Association of ACS application with clinical and nutritional outcomes

In terms of nutrition outcomes, compared with non-ACS group, single-course ACS group had higher proportion of complete breastfeeding in feeding pattern in GA < 28 weeks, and had significant earlier application of amino acid and enteral nutrition in 28 weeks ≤ GA < 32 weeks. We also found repeated course ACS group had larger proportion of mixed feeding, and less 100% preterm formula milk in feeding pattern in 28 weeks ≤ GA < 32 weeks (Table 1). After adjustment for potential confounding factors of birth demographics and respiratory-related indicators, single-course ACS showed positive relationship to the introduction of amino acid and enteral nutrition in 28 weeks ≤ GA < 32 weeks, however, repeated course ACS showed no significant correlation with nutritional outcomes (Table 2).

**Table 1:** Comparison of clinical and nutritional outcomes of VLBW preterm infants between different ACS courses

Variable	GA < 28 weeks (*n* = 66)				28 weeks ≤ GA < 32 weeks (*n* = 591)				32 weeks ≤ GA < 37 weeks (*n* = 184)			
	Non-ACS (*n* = 39)	Single- course ACS (*n* = 11)	Repeated course ACS (*n* = 16)	*P*	Non-ACS (*n* = 315)	Single- course ACS (*n* = 111)	Repeated course ACS (*n* = 165)	*P*	Non-ACS (*n* = 102)	Single- course ACS (*n* = 40)	Repeated course ACS (*n* = 42)	*P*
Cesarean section	7(17.9)	3(27.3)	4(25.0)	0.731	167(53.0) ^*a*^	50(54.5) ^*a*^	116(70.3) ^*b*^↑	< 0.001*	80(78.4)	30(75.0)	36(85.7)	0.460
Male	26(66.7)	8(72.7)	12(75.0)	0.804	211(67.0)	69(62.2)	99(60.0)	0.100	39(38.2)	17(42.5)	18(42.9)	0.829
Birthweight, g	1070(1000–1180)	1010(960–1055)	1000(855–1082)	0.085	1290(1150–1400)	1300(1200–1400)	1300(1150–1385)	0.174	1385(1258–1440.)	1345 (1250–1457)	1375(1278–1441)	0.420
5 min APGAR> 7	39(88.6)	13(92.9)	17(77.3)	0.697	283(89.8)	106(95.5)	155(93.9)	0.073	96(94.1)	39(97.5)	42(100.0)	0.187
Intubation ventilation, days	7(0–17)	2(0–6)	4(0–23)	0.346	2 (0–5) ^*a*^	0(0–2) ^*b*^↓	0(0–4) ^*b*^↓	< 0.001*	0(0–0)	0(0–1)	0(0–0)	0.016*
Non-invasive ventilation, days	4(1–20)	6(2–19)	6(1–13)	0.860	3(0–6)	2 (0–6)	3(0–6)	0.276	0(0–2)	1(0–3)	1(0–3)	0.379
NEC	3(7.7)	1(9.1)	1(6.3)	0.927	18(5.7)	5(4.5)	6(3.6)	0.592	5(4.9)	3(7.5)	0(0.0)	0.177
Death	0(0.0)	2(18.2)	2(12.5)	0.038*	16(5.1)	6(5.4)	8(4.8)	0.974	4(3.9)	2(5.0)	0(0.0)	0.300
Amino acid introduction, DOL	1(0–1)	0(0–1)	1(1–1)	0.078	1(1–1) ^*a*^	1(0–1) ^*b*^↓	1(0–1) ^*ab*^	0.001*	1(1–1)	1(0–1)	1(0–1)	0.997
Duration of amino acid, days	41(28–52)	37(12–43)	35(19–60)	0.425	28(19–38)	26(19–37)	28(20–38)	0.702	19(11–26)	20(12–25)	20(15–29)	0.442
Lipid introduction, DOL	2(1–6)	2(1–3)	2(1–7)	0.423	2(1–4)	2(1–3)	2(1–3)	0.041*	2(1–4)	2(1–4)	1(1–5)	0.961
Duration of lipid, days	29(19–43)	27(7–38)	26(12–58)	0.552	23(14–34)	21(13–31)	23(15–31)	0.405	14(9–21)	17(12–23)	17(10–23)	0.013*
Feeding pattern				0.019*				0.001*				0.249
Complete breastfeeding	0(0.0) ^*a*^	2(18.2)^*b*^↑	0(0.0) ^*ab*^		8(2.5)	5(4.5)	8(4.8)		4(3.9)	3(7.5)	4(9.5)	
Mixed feeding	3(7.7)	1(9.1)	0(0.0)		13(4.1) ^*a*^	10(9.0) ^*ab*^	29(17.6) ^*b*^↑		5(4.9)	4(10.0)	6(14.3)	
Formula feeding	36(92.3)	8(72.7)	16(100.0)		294(93.3) ^*a*^	96(86.5) ^*ab*^	128(77.6) ^*b*^↓		93(91.2)	33(82.5)	32(76.2)	
Start enteral nutrition, DOL	4(2–7)	2(2–6)	5(3–9)	0.071	4.0(2.0–5.0) ^*a*^	2.0(2.0–4.0)^*b*^↓	3.0(2.0–5.0)^*ab*^	< 0.001*	3.0(2.0–5.0)	2.0(2.0–3.0)	2.0(1.0–4.0)	0.471
Enteral feeding volumes of 150 mL·kg^-1^·d^-1^, DOL	44(32–58)	42(39–56)	44 (23–54)	0.828	35(25–46)	31 (24–43)	35 (24–46)	0.085	21(13–26)	21(15–28)	25(18–30)	0.840

VLBW, very-low-birth-weight; ACS, Antenatal corticosteroids; GA, gestational age; DOL, day of life; NEC, necrotizing enterocolitis

Data are median (interquartile range) or n (%) unless otherwise specified

Data are analyzed by Kruskal-Wallis *H* test or *χ* 2 test. Mann-Whitney test is used for post hoc analysis. * *P* < 0.05. ^ab^post hoc analysis

**Table 2:** Multivariate analysis of ACS courses association with nutritional outcomes in VLBW infants stratified by GA

Variable	Total		28 weeks ≤ GA < 32 weeks	
	Single-course ACS	Repeated course ACS	Single-course ACS	Repeated course ACS
Amino acid introduction^a^	−0.11 (− 0.70, − 0.15) *	−0.05 (−0.44,0.04)	−0.09 (−0.71, −0.04) *	−0.04 (−0.44,0.14)
Lipid introduction^b^	−0.04 (1.42,0.38)	−0.05 (− 1.35,0.23)	− 0.02 (−1.46,0.76)	− 0.05 (− 1.57,0.34)
Enteral nutrition introduction^c^	−0.08 (−1.35, − 0.11) *	− 0.04 (− 0.87,0.22)	−0.09 (− 1.52, − 0.01) *	− 0.05 (− 1.08,0.22)
Enteral feeding volumes of 150 mL·kg^− 1^·d^-1d^	− 0.03 (−3.70,0.94)	0.006 (− 1.89,2.30)	− 0.03 (− 4.22,1.50)	0.009 (− 2.24,2.84)

Data are adjusted linear regression coefficient (95% CI), refer to non-ACS

*VLBW* very-low-birth-weight, *ACS* Antenatal corticosteroids, *GA* gestational age

*multiple linear regression shows significant outcomes. No significant outcomes were found in GA < 28 weeks or 32 weeks ≤ GA < 37 weeks group

^a^ adjusted for GA, 5 min APGAR score

^b^ adjusted for GA, 5 min APGAR score and amino acid introduction

^c^ adjusted for GA, birth weight, cesarean section, lipid introduction and amino acid introduction

^d^ adjusted for GA, birth weight, cesarean section, lipid introduction, amino acid introduction and feeding pattern

Pertaining to clinical outcomes, both single-course ACS group and repeated course ACS group had shorter time on intubation ventilation support compared with non-ACS group. Repeated course group had the highest rate of cesarean section among the three groups only in 28 weeks ≤ GA < 32 weeks. No differences in clinical outcomes were found between single-course ACS group and repeated course ACS group (Table 1).

### Association of ACS application with postnatal weight growth

**Fig. 1:**
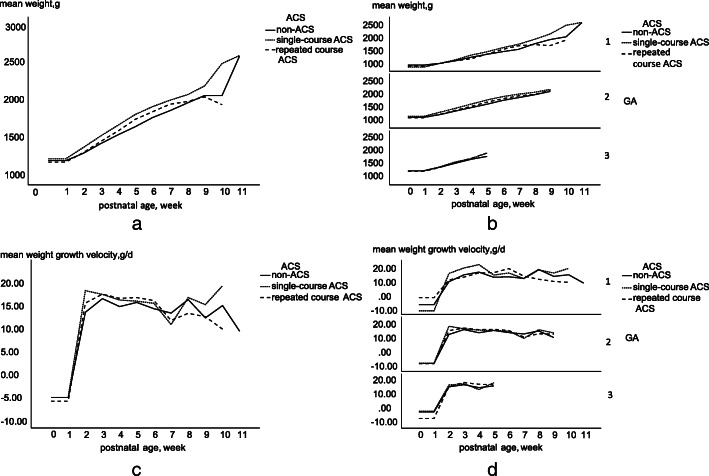
Curves trajectories of weight-growth and weight-growth velocity in the VLBW infants according to ACS regimens. VLBW, very-low-birth-weight; ACS, antenatal corticosteroids; GA, gestational age. Data are mean weight (**a** and **b**) or mean weight-growth velocity (**c** and **d**). **a**: Curve trajectories of mean weight in total VLBW preterm infants. **b**: Curve trajectories of mean weight in total VLBW preterm infants stratified by GA. **c**: Curve trajectories of mean weight-growth velocity in total VLBW preterm. **d**: Curve trajectories of mean weight-growth velocity in total VLBW preterm stratified by GA. GA: 1 = GA < 28 weeks, 2 = 28 weeks ≤ GA < 32 weeks, 3 = 32 weeks ≤ GA < 37 weeks. Data were collected until 37 postmenstrual age

The weight growth trajectories of VLBW preterm infants were shown in Fig. 1. The trajectories of mean weight (shown in Fig. 1. B) and weight growth velocity (shown in Fig. 1. D) were also examined separately by different birth GA. Differences in the weight-growth curve trajectories were assessed in Table 3.

**Table 3:** Relationship between ACS with weight changes during hospitalization in VLBW preterm infants

Variable	Unadjusted coefficient	Adjusted coefficient ^a^
Total		
Postnatal age, week	45.52(40.91,50.12) *	74.23(56.03,92.42) *
Non-ACS	0.00(Reference)	0.00(Reference)
Single-course	23.54(−9.32,56.39)	1.54(− 29.76,32.84)
Repeated course	− 19.29(− 48.41,9.82)	− 12.61(− 40.30,15.06)
Time* single-course	18.24(10.07,26.42) *	14.75(6.02,23.49) *
Time* repeated course	10.12(2.94,17.31) *	5.48(−2.24,13.21)
Repeated course	0.00(Reference)	0.00(Reference)
Single-course	42.83(5.94,79.71) *	14.15(−20.38,48.70)
Time* single-course	8.12(−1.02,17.26)	9.27(−0.41,18.96)
GA < 28 weeks		
Postnatal age, week	34.10(19.85,48.35) *	53.59(−21.00,128.20)
Non-ACS	0.00(Reference)	0.00(Reference)
Single-course	−81.72(−201.53,38.09)	−83.94(− 211.27,43.38)
Repeated course	−38.34(−138.91,62.23)	3.73(−98.58,106.05)
Time* single-course	22.00(−6.99,50.98)	20.31(−13.07,53.70)
Time* repeated course	4.21(−19.85,28.27)	4.68(−21.17,30.54)
Repeated course	0.00(Reference)	0.00(Reference)
Single-course	−43.38(−179.30,92.55)	−87.68(− 232.75,57.38)
Time* single-course	17.79(−15.27,50.85)	15.63(−21.84,53.10)
28 weeks ≤ GA < 32 weeks		
Postnatal age, week	43.27(37.93,48.61) *	73.43(51.71,95.15) *
Non-ACS	0.00(Reference)	0.00(Reference)
Single-course	31.99(−4.93,68.90)	10.90(−25.09,46.90)
Repeated course	−14.48(−46.59,17.63)	−14.67(−46.41,17.06)
Time* single-course	19.99(10.51,29.46) *	15.71(5.54,25.88) *
Time* repeated course	10.55(2.36,18.73) *	5.26(−3.66,14.20)
Repeated course	0.00(Reference)	0.00(Reference)
Single-course	46.47(5.51,87.42) *	25.57(−14.02,65.17)
Time* single-course	9.43(−1.05,19.92)	10.44(−0.81,21.69)
32 weeks ≤ GA < 37 weeks^b^		

Point estimates and 95% *CI* of differences in mean weight or weight growth rate are shown

*VLBW* very-low-birth-weight, *ACS* Antenatal corticosteroids, *GA* gestational age

*Significantly different from reference group

^a^ Adjusted for sex, feeding pattern, cesarean section rate, time to start enteral nutrition, 5 min APGAR score, GA, multiple birth, amino acid and lipid introduction, duration of amino acid and lipid

^b^ Iteration is terminated but convergence has not been achieved. Validity of the model fit is uncertain

All data were collected until 37 postconceptional age or at discharge

In unadjusted model, single-course ACS group had a greater average weight over time compared with the repeated course ACS group. Also, single-course ACS and repeated course ACS groups demonstrated higher weight increase rate over time compared with the non-ACS group. After adjusting for confounding factors of birth demographics, mode of delivery, nutritional and respiratory-related indicators, single-course ACS group had higher weight increase rate compared with non-ACS group, however, there were no differences in mean weight among groups. Finally, identical growth advantage for single-course ACS were observed in VLBW preterm infants born at 28–32 weeks’ gestation. No differences in mean weight and weight growth rate among non-ACS, single-course and repeated course ACS treatments were found in other GA group in multivariable analysis.

## Discussion

Previous studies showed unclear effects of ACS on postnatal growth and feeding in VLBW preterm infants. The present study showed a close relationship between single-course ACS application and the remarkable improvement in terms of amino acid introduction, enteral nutrition introduction, and weight increase rate in VLBW infants born at 28–32 GA. Additionally, both single and repeated course of ACS regimens had shorter intubated ventilation time compared to non-ACS regimen.

Single-course ACS positively correlated with earlier application of amino acid and enteral nutrition with VLBW preterm infants with 28–32 GA. On the contrary, repeated course ACS did not show any association in all GA groups. Previous studies showed that ACS improved the bowel maturation in a manner similar to the lung, that is due to its gastrointestinal tract maturation enhancement effect and anti-inflammatory properties [25, 26]. Animal researches [18, 19] and human studies [20] suggested that ACS could promote the secretion of gut hormones and the growth of the gastrointestinal tract. Enteral nutrition could be introduced when there was bowel motility. The bowel motility patterns were abnormal and incompletely developed before 28 GA [27]. This may be the reason why we did not observe the difference between groups before 28 GA. Motilin receptors and cyclic motilin release are present after 32 GA [27]. Therefore, we speculated that the effect of ACS exposure on the time to start enteral feeding became insignificant after 32 GA. A study [28] published in a Chinese journal has the similar view. The IUGR rate in repeated course ACS was higher compared with single-course ACS. When stratified according to IUGR or non-IUGR, the above conclusions were consistent in the non-IUGR group (supplementary material).

The results from the present study showed that although there was no difference in birthweight among non-ACS, single-course ACS and repeated course ACS groups, postnatal growth rate was found to be different. Single-course ACS group had higher weight increase rate in infants born at 28–32 GA, however, repeated course ACS did not improve the average weight and weight increase rate during hospitalization after adjusting for sex, cesarean section rate, 5 min APGAR score, GA, multiple birth and nutritional factors. Prior animal and human studies have suggested that multiple repeated ACS may impair fetal growth [29, 30] or have no effects [11, 12, 14], Some studies found no association between multiple repeated ACS with adverse effects on growth at discharge [13], in 2–3 years of age or in adolescence [14–17]. These studies measured weight at birth or at single point time after a period of time, while we managed to collect the longitudinal postnatal measurement. A retrospective study reported that ACS did not affect mean weight in the nursery [31], which was largely consistent with our research. Another study [32] focused on both mean weight gain and weight growth rate. The researchers measured weight at birth and weekly for 4 weeks or until discharge, and they found infants before 32 GA exposed to repeated course ACS demonstrated postnatal growth acceleration (single-course ACS was not concluded in their study), which was consistent with our results before adjusting for postnatal clinical and nutritional status. Considering the above factors, we only found that single-course ACS had higher weight growth rate related to non-ACS.

VLBW preterm infants could experience ‘catch-up’ growth after birth over the weeks and months [33]. Interestingly, the negative effects of ACS on birthweight [29] may not be observed after birth in a period of time [14]. We observed a more rapidly increase in weight growth rate in the group exposed to single-course ACS at 28–32 GA. This meant that the VLBW preterm infants may recovery from the potential growth inhibitory effects of ACS shortly after birth [32]. The recovery was presumably related to the physiology and nutrition around the time of birth, especially in the repeated course ACS group. The “GA window” (28–32 GA) further illustrated that this recovery may be due to the effects of ACS on the gastrointestinal tract. Of note, fast growth catchup does not necessarily imply a better long-term outcome [34, 35]. The mechanism of influences of different courses ACS on the gastrointestinal tract is still unclear. Therefore, we expect more studies providing evidence for it, as well as the impact on catching growth.

Both single-course and repeated course ACS were associated with shorter time of intubation ventilation in infants born at 28–32 GA, which was consistent with previous researches [14, 16, 36–38]. Moreover, the effects of ACS on clinical outcomes in preterm infants predominately depended on GA, that is mainly because of different stages of fetal organ development seen in various GA stages [39, 40]. In this study, the duration of intubation ventilation was not different among non-ACS, single-course ACS and multi-course ACS groups in infants < 28 GA. Observational studies have shown inconsistent findings in infants at the lowest gestations. A multicenter study [41] of infants < 29 weeks’ gestation found a higher incidence of respiratory complications in infants exposed to ACS than in those not exposed. However, another large multicenter observational study [42] did not find a difference in the incidence of respiratory complications between the exposed and unexposed ACS groups. The Cochrane review [14, 43] subgroup analysis (4 randomized controlled trials, 102 infants) of those infants < 28 GA also found that the incidence of RDS exposed to ACS did not differ compared to infants who were not exposed. Randomized controlled trials with adequate sample sizes are needed to explain the role of ACS in extremely preterm infants.

A major strength of this study was that we continuously measured the weight gain of VLBW preterm infants during hospitalization, compared with previous studies focusing on separate time points. Our study focused on nutrition-related outcomes during hospitalization, while it was less frequently discussed in previous studies. We found that single-course ACS treatment had the greatest benefit for VLBW infants born at 28–32 weeks, suggesting that more cautions are needed when expanding the use of ACS in clinical practice.

Limitations in the study included that it was a retrospective study in design, with some potentially confounders unmeasured or uncollected, such as specific data of ACS use, maternal history and sociodemographic characteristics. Furthermore, some parents required an early discharge of their infants subsequently some related clinical outcomes were unclear. We did not make a detailed distinction between one repeat course or multiple weekly repeat course in this study, since the questionnaire was designed and the data were collected during 2011–2014, when there were no guideline recommendations for the use of repeated course ACS. The ACS used in these hospitals was dexamethasone, therefore the final conclusions could not represent other medicine treatment regimen.

## Conclusions

In summary, single-course ACS treatment was positively related to parenteral and enteral feeding, as well as weight growth in VLBW preterm infants born at 28–32 weeks’ gestation. However, no associations of repeated course ACS with feeding and growth were observed, which indicates that more attention are needed to pay when expanding the use of ACS in clinical practice.

## Supplementary Information

**Additional file 1.**

## Data Availability

The datasets generated during the current study are not publicly available due to this study was a multicenter study, the agencies were keeping the data confidential but are available from the corresponding author on reasonable request.

## Abbreviations

ACS: Antenatal corticosteroids
RDS: Respiratory distress syndrome
GA: Gestational age
VLBW: Very-low-birthweight
NEC: Necrotizing enterocolitis
NICU: Neonatal intensive care units
IUGR: Intrauterine growth retardation

## References

[1] Kemp MW, Schmidt AF, Jobe AH (2019). Optimizing antenatal corticosteroid therapy. Semin Fetal Neonatal Med.

[2] Sweet DG, Carnielli V, Greisen G, Hallman M, Ozek E, Te Pas A (2019). European consensus guidelines on the Management of Respiratory Distress Syndrome - 2019 update. Neonatology..

[3] G. C. Liggins M, FRCOG, R. N. Howie, MB, MRACP. A Controlled Trial of Antepartum Glucocorticoid Treatment for Prevention of the Respiratory Distress Syndrome in Premature Infants. Pediatrics. 1972;50:515–525. 4561295

[4] GC L. Premature delivery of foetal lambs infused with glucocorticoids. J Endocrinol 1969;45:515–523. 10.1677/joe.0.0450515 5366112

[5] Vermillion ST, Bland ML, Soper DE (2001). Effectiveness of a rescue dose of antenatal betamethasone after an initial single course. Am J Obstet Gynecol.

[6] ACOG. American College of Obstetricians and Gynecologists (2017). ACOG committee opinion No. 713: antenatal corticosteroid therapy for fetal maturation. Obstet Gynecol.

[7] Groom KM (2019). Antenatal corticosteroids after 34weeks' gestation: do we have the evidence?. Semin Fetal Neonatal Med.

[8] Bonanno C, Wapner RJ (2012). Antenatal corticosteroids in the management of preterm birth: are we back where we started?. Obstet Gynecol Clin N Am.

[9] Haram K, Mortensen JH, Magann EF, Morrison JC (2017). Antenatal corticosteroid treatment: factors other than lung maturation. J Matern Fetal Neonatal Med.

[10] French NP, Hagan R, Evans SF, Godfrey M, Newnham JP (1999). Repeated antenatal corticosteroids: size at birth and subsequent development. Am J Obstet Gynecol.

[11] C. McEvoy, D. Schilling, D. Peters, C. Tillotson, P. Spitale, L. Wallen, et al. Respiratory compliance in preterm infants after a single rescue course of antenatal steroids: a randomized controlled trial. Am J Obstet Gynecol, 202 (2010) 544.e1–9. 10.1016/j.ajog.2010.01.038 PMC2878893 20227053

[12] Garite TJ, Kurtzman J, Maurel K, Clark R (2009). Impact of a ‘rescue course’ of antenatal corticosteroids: a multicenter randomized placebo-controlled trial. Obstetrix Collaborative Research Network. Am J Obstet Gynecol.

[13] Crowther CA, Haslam RR, Hiller JE, Doyle LW, Robinson JS (2006). Neonatal respiratory distress syndrome after repeat exposure to antenatal corticosteroids: a randomised controlled trial. Lancet.

[14] Roberts D, Brown J, Medley N, Dalziel SR (2017). Antenatal corticosteroids for accelerating fetal lung maturation for women at risk of preterm birth. Cochrane Database Syst Rev.

[15] Doyle LW, Ford GW, Rickards AL, Kelly EA, Davis NM, Callanan C (2000). Antenatal corticosteroids and outcome at 14 years of age in children with birth weight less than 1501 grams. Pediatrics..

[16] Wapner RJ, Sorokin Y, Thom EA, Johnson F, Dudley DJ, Spong CY (2006). Single versus weekly courses of antenatal corticosteroids: evaluation of safety and efficacy. Am J Obstet Gynecol.

[17] Crowther CA, Doyle LW, Haslam RR, Hiller JE, Harding JE, Robinson JS (2007). Outcomes at 2 years of age after repeat doses of antenatal corticosteroids. N Engl J Med.

[18] Doell RG, Kretchmer N (1964). Intestinal invertase: precocious development of activity after injection of hydrocortisone. Science..

[19] Koldovski O, Sunshine P (1970). Effect of cortisone on the developmental pattern of the neutral and the acid hgalactosidase of the small intestine of the rat. Biochem J.

[20] Villa M, Menard D, Semenza G, Mantei N (1992). The expression of lactase enzymatic activity and mRNA in human fetal jejunum. Effect of organ culture and of treatment with hydrocortisone. FEBS Lett.

[21] de Waard M, Li Y, Zhu Y, Ayede AI, Berrington J, Bloomfield FH (2019). Time to full enteral feeding for very low-birth-weight infants varies markedly among hospitals worldwide but may not be associated with incidence of necrotizing Enterocolitis: the NEOMUNE-NeoNutriNet cohort study. JPEN J Parenter Enteral Nutr.

[22] Agostoni C, Buonocore G, Carnielli V, De Curtis M, Darmaun D, Decsi T (2010). Enteral nutrient supply for preterm infants: commentary from the European Society of Paediatric Gastroenterology, Hepatology and nutrition committee on nutrition. J Pediatr Gastroenterol Nutr.

[23] Facco F, Louis J, Knavert MP, Izci Balserak B. Chapter 157 - Sleep-Disordered Breathing in Pregnancy. In: Kryger M, Roth T, Dement WC, editors. Principles and Practice of Sleep Medicine (Sixth Edition): Elsevier; 2017.p.1540–6.e4.

[24] Patel AL, Engstrom JL, Meier PP, Kimura RE (2005). Accuracy of methods for calculating postnatal growth velocity for extremely low birth weight infants. Pediatrics..

[25] Bauer CR, Morrison JC, Poole WK, Korones SB, Boehm JJ, Rigatto H (1984). A decreased incidence of necrotizing enterocolitis after prenatal glucocorticoid therapy. Pediatrics..

[26] Thompson AM, Bizzarro MJ (2008). Necrotizing Enterocolitis in Newborns Pathogenesis, Prevention and Management. Drugs.

[27] Berseth CL (1996). Gastrointestinal motility in the neonate. Clin Perinatol.

[28] Menghua W, Yannan Z, Zheng Z. [Analysis of factors influencing feeding intolerance in preterm infants with different gestational ages]. Zhongnan Journal of Medical Sciences. 2017;DOI:10.15972/j.cnki.43-1509/r.2017.02.013. 26.[in Chinese].

[29] Norberg H, Stålnacke J, Heijtz RD, Smedler A-C, Nyman M, Forssberg H (2011). Antenatal corticosteroids for preterm birth: dose-dependent reduction in birthweight, length and head circumference. Acta Paediatr.

[30] Aghajafari F, Murphy K, Matthews S, Ohlsson A, Amankwah K, Hannah M (2002). Repeated doses of antenatal corticosteroids in animals: a systematic review. Am J Obstet Gynecol.

[31] Thorp JA, Jones PG, Peabody JL, Knox E, Clark RH (2002). Effect of antenatal and postnatal corticosteroid therapy on weight gain and head circumference growth in the nursery. Obstet Gynecol.

[32] Battin M, Bevan C, Harding J (2012). Growth in the neonatal period after repeat courses of antenatal corticosteroids: data from the ACTORDS randomised trial. Arch Dis Child Fetal Neonatal Ed.

[33] Fattal-Valevski A, Toledano-Alhadef H, Leitner Y, Geva R, Eshel R, Harel S (2009). Growth patterns in children with intrauterine growth retardation and their correlation to neurocognitive development. J Child Neurol.

[34] Bruschettini M, van den Hove DL, Gazzolo D, Steinbusch HW, Blanco CE (2006). Lowering the dose of antenatal steroids: the effects of a single course of betamethasone on somatic growth and brain cell proliferation in the rat. Am J Obstet Gynecol.

[35] Gluckman PD, Hanson MA (2004). Living with the past: evolution, development, and patterns of disease. Science..

[36] Murphy K, Aghajafari F (2003). Single versus repetitive courses of corticosteroids: what do we know?. Clin Obstet Gynecol.

[37] Blickstein I, Shinwell ES, Lusky A, Reichman B, Israel NN (2005). Plurality-dependent risk of respiratory distress syndrome among very-low-birth-weight infants and antepartum corticosteroid treatment. Am J Obstet Gynecol.

[38] Quist-Therson EC, Myhr TL, Ohlsson A (1999). Antenatal steroids to prevent respiratory distress syndrome: multiple gestation as an effect modifier. Acta Obstet Gynecol Scand.

[39] Romejko-Wolniewicz E, Teliga-Czajkowska J, Czajkowski K (2014). Antenatal steroids: can we optimize the dose?. Curr Opin Obstet Gynecol.

[40] Davidoff MJ, Dias T, Damus K, Russell R, Bettegowda VR, Dolan S (2006). Changes in the gestational age distribution among U.S. singleton births: impact on rates of late preterm birth, 1992 to 2002. Semin Perinatol.

[41] Wong D, Abdel-Latif M, Kent A (2014). NICUS Network Antenatal steroid exposure and outcomes of very premature infants: a regional cohort study. Arch Dis Child Fetal Neonatal Ed.

[42] Travers CP, Carlo WA, McDonald SA, Das A, Bell EF, Ambalavanan N, et al. Mortality and pulmonary outcomes of extremely preterm infants exposed to antenatal corticosteroids. Am J Obstet Gynecol. 2018; 218 1:130 e1- e13. 10.1016/j.ajog.2017.11.554 PMC5842434 29138031

[43] Roberts D, Dalziel S (2006). Antenatal corticosteroids for accelerating fetal lung maturation for women at risk of preterm birth. Cochrane Database Syst Rev.

