# Supplementary material for: Single-course antenatal corticosteroids is related to faster growth in very-low-birth-weight infant
Source: BMC Pregnancy Childbirth. 2021 Jan 12;21:50. doi: 10.1186/s12884-020-03510-w (PMC7801876; doi:10.1186/s12884-020-03510-w)
Supplement: Supplementary file 1 — Additional file 1. [file 12884_2020_3510_MOESM1_ESM.docx]

# Supplementary material: Analyses in Non-IUGR/IUGR VLBW Infants

## Expanded Methods

Sensitivity analyses were performed to evaluate the association of single-course or repeated course ACS with nutritional outcomes and postanal growth in IUGR infants or non-IUGR infants. All analyses were stratified by GA. Multiple linear regression was used to analyze the association of ACS with nutritional outcomes and clinical outcomes. Multilevel model was used to analyze the association of ACS with postanal weight growth. The coefficients for ACS were related to differences in mean weight. The coefﬁcients for the interactions between the ACS and time could be interpreted to indicate whether differences in mean weight varied over time (growth rate).

## Expanded Results

Layered according to non-IUGR or IUGR, single-course ACS showed positive association with amino acid introduction and enteral nutrition introduction. However, repeated course ACS showed no significant correlation with nutritional and clinical outcomes in non-IUGR infants born at 28–32 GA. No significant correlations were found in IUGR group as shown in eTable 1 and eTable 2.

Layered according to non-IUGR or IUGR, in unadjusted model, compared with non-ACS group, no differences in mean weight were found in both single-course and repeated course ACS groups. The single-course and repeated course ACS groups demonstrated higher growth rate of weight over time. After adjusting for sex, feeding pattern, cesarean section rate, time to start enteral nutrition, 5min APGAR score, GA, multiple birth, amino acid introduction, lipid introduction, duration of amino and lipid in model 2, there were no significant differences in mean weight among the three groups. Single course group had higher growth rate of weight over time compared with non-ACS group (coefficient >0). The outcomes were similar to eTable 4 in non-IUGR VLBW infants born at 28–32 GA. No differences in mean weight and weight growth rate were found in other GA group and in all IUGR infants though the validity of the model fit is uncertain in IUGR group as shown in eTable 3 and eTable 4.

eTable 1 Association of single- and repeated course ACS with nutritional and clinical outcomes in non-IUGR VLBW preterm infants

| GA, weeks | Amino introduction^a^ | Lipid introduction^b^ | Enteral nutrition introduction^c^ | time to enteral feeding volumes of 120 mL·kg^-1^·d^-1d^. | time to enteral feeding volumes of 150 mL·kg^-1^·d^-1d^. | Discharged weight^e^ |
| --- | --- | --- | --- | --- | --- | --- |
| Total | | | | | | |
| Single-course | −0.11  (−0.77, −0.14) * | −0.05  (−1.76,0.40) | −0.10  (−1.55, −0.17) * | −0.02  (−3.33,1.65) | −0.03  (−4.15,1.54) | 0.006  (−47.33,60.70) |
| Repeated course | −0.04  (−0.42,0.14) | −0.05  (−1.60,0.30) | −0.04  (−0.92,0.31) | −0.01  (−2.55,1.97) | 0.009  (−2.27,2.90) | 0.02  (−30.10,66.28) |
| GA<28 | | | | | | |
| Single-course | −0.21  (−1.90,0.16) | −0.06  (−4.08,2.16) | −0.04  (−2.71,1.92) | −0.10  (−17.97,8.51) | −0.09  (−21.05,10.45) | −0.008  (−239.98,215.96) |
| Repeated course | −0.05  (−1.09,0.73) | −0.03  (−3.19,2.27) | 0.15  (−0.77,3.28) | −0.13  (−16.04,5.04) | −0.06  (−15.75,9.34) | −0.02  (−219.93,158.73) |
| 28-32 GA | | | | | | |
| Single-course | −0.10  (−0.75, −0.04) * | −0.03  (−1.68,0.71) | −0.10  (−1.55, −0.05) * | −0.02  (−3.40,1.84) | −0.03  (−4.15,1.73) | 0.01  (−46.69,69.77) |
| Repeated course | −0.03  (−0.43,0.20) | −0.04  (−1.60,0.53) | −0.05  (−1.10,0.25) | 0.00  (−2.42,2.40) | 0.02  (−2.01,3.40) | 0.01  (−38.99,66.70) |
| GA>32 | | | | | | |
| Single-course | −0.28  (−1.32,0.17) | −0.01  (−2.92,2.73) | −0.08  (−3.22,1.97) | 0.08  (−5.39,8.32) | 0.07  (−7.18,10.21) | 0.05  (−311.65,389.67) |
| Repeated course | −0.28  (−1.16,0.11) | −0.02  (−2.35,2.04) | −0.24  (−3.43,0.43) | 0.35  (−0.79,11.99) | 0.30  (−2.23,13.99) | 0.40  (−129.30,563.20) |

VLBW, very-low-birth-weight; ACS, Antenatal corticosteroids; GA, gestational age; IUGR, Intrauterine growth retardation.

Data are adjusted linear regression coefficient (95% CI), refer to non-ACS.

*multiple linear regression shows significant differences.

^a^ adjusted for GA, 5min APGAR score.

^b^ adjusted for GA, 5min APGAR score and amino acid introduction.

^c^ adjusted for GA, birth weight, cesarean section, lipid introduction and amino acid introduction.

^d^ adjusted for GA, birth weight, cesarean section, lipid introduction, amino acid introduction and feeding pattern.

^e^ adjusted for GA, birth weight, regain birth weight, extra oxygen supply, intubation ventilation, inpatient time, amino acid introduction, duration of amino acid, probiotics and lipid introduction.

eTable 2 Association of single- and repeated course ACS with nutritional and clinical outcomes in IUGR VLBW infants

| GA, weeks | Amino introduction^a^ | Lipid introduction^b^ | Enteral nutrition introduction^c^ | time to enteral feeding volumes of 120 mL·kg^-1^·d^-1d^. | time to enteral feeding volumes of 150 mL·kg^-1^·d^-1d^. | Discharged weight^e^ |
| --- | --- | --- | --- | --- | --- | --- |
| Total | | | | | | |
| Single-course | −0.08  (−0.88,0.24) | −0.01  (−1.70,1.39) | −0.01  (−1.50,1.23) | −0.01  (−3.46,2.76) | −0.04  (−4.59,2.20) | 0.10  (−14.52,211.40) |
| Repeated course | −0.12  (−0.89,0.06) | −0.02  (−1.50,1.06) | −0.06  (−1.65,0.68) | −0.04  (−3.83,1.66) | −0.02  (−3.52,2.48) | 0.12  (−5.06,191.72) |
| GA<28, IUGR | | | | | | |
| Single-course |  |  |  |  |  |  |
| Repeated course |  |  |  |  |  |  |
| 28-32 GA | | | | | | |
| Single-course | −0.007  (−1.12,1.06) | 0.04  (−1.86,2.82) | 0.14  (−3.27,8.65) | −0.02  (−19.23,17.06) | −0.005  (−18.81,18.15) | 0.07  (−259.15,529.82) |
| Repeated course | −0.25  (−0.81,0.06) | −0.15  (−1.71,0.40) | −0.06  (−3.04,2.04) | −0.006  (−7.76,7.49) | −0.04  (−8.74,6.80) | 0.25  (5.87,379.05) |
| GA>32 | | | | | | |
| Single-course | −0.09  (−1.06,0.28) | −0.04  (−2.37,1.43) | −0.02  (−1.43,1.15) | −0.02  (−2.96,2.13) | −0.07  (−4.22,1.59) | 0.10  (−43.03,204.89) |
| Repeated course | −0.07  (−0.98,0.38) | 0.01  (−1.80,2.03) | −0.08  (−1.94,0.66) | −0.05  (−3.42,1.75) | 0.02  (−2.45,3.44) | 0.06  (−77.68,171.54) |

VLBW, very-low-birth-weight; ACS, Antenatal corticosteroids; GA, gestational age; IUGR, Intrauterine growth retardation.

Data are adjusted linear regression coefficient (95% CI), refer to non-ACS.

*multiple linear regression shows significant differences. Blank cells show data which couldn’t computed.

a adjusted for GA, 5min APGAR score.

b adjusted for GA, 5min APGAR score and amino acid introduction.

c adjusted for GA, birth weight, cesarean section, lipid introduction and amino acid introduction.

d adjusted for GA, birth weight, cesarean section, lipid introduction, amino acid introduction and feeding pattern.

e adjusted for GA, birth weight, regain birth weight, extra oxygen supply, intubation ventilation, inpatient time, amino acid introduction, duration of amino acid, probiotics and lipid introduction.

eTable 3 The relationship between ACS with weight changes during hospitalization in non-IUGR VLBW Infants

| Variable | non-IUGR | |
| --- | --- | --- |
|  | Unadjusted coefficient | Adjusted coefficient^a^ |
| Total | | |
| Postnatal age, week | 47.51(42.16,52.87) * | 74.44(53.04,95.85) * |
| ACS use, course |  |  |
| Non-ACS | 0.00(Reference) | 0.00(Reference) |
| Single-course | 11.56(−23.25,46.38) | −15.84(−46.50,14.81) |
| Repeated course | −6.41(−37.72,24.89) | −16.14(−43.58,11.29) |
| Time* single-course | 18.49(9.16,27.82)* | 15.53(5.55,25.51) * |
| Time* repeated course | 8.72(0.43,17.01) * | 4.55(−4.37,13.49) |
| Repeated course | 0.00(Reference) | 0.00(Reference) |
| Single-course | 17.9(−21.08,57.04) | 0.298(−33.50,34.10) |
| Time* single-course | 9.76(−0.65,20.19) | 10.97(−0.09,22.04) |
| GA<28weeks | | |
| Postnatal age, week | 36.69(21.99,51.39) * | 56.857(−19.60,133.32) |
| ACS use, course |  |  |
| Non-ACS | 0.00(Reference) | 0.00(Reference) |
| Single-course | −85.74(−206.52,35.03) |  |
| Repeated course | −41.67(−143.19,59.83) | −0.40(−104.06,103.25) |
| Time* single-course | 22.49(−7.12,52.10) | 20.56(−13.66,54.79) |
| Time* repeated course | 4.28(−20.32,28.89) | 4.64(−21.96,31.25) |
| Repeated course | 0.00(Reference) | 0.00(Reference) |
| Single-course | −44.06(−180.71,92.58) | −91.01(−237.34,55.31) |
| Time* single-course | 18.25(−15.47,51.88) | 15.92(−22.37,54.21) |
| 28weeks≤GA<32weeks | | |
| Postnatal age, week | 45.38(39.65,51.11) * | 73.47(50.72,96.23) * |
| ACS use, course |  |  |
| Non-ACS | 0.00(Reference) | 0.00(Reference) |
| Single-course | 13.48(−20.79,47.75) | −5.77(−38.01,26.46) |
| Repeated course | −4.98(−36.08,26.10) | −9.95(−39.43,19.52) |
| Time* single-course | 18.87(8.99,28.76)* | 14.77(4.18,25.35) * |
| Time* repeated course | 8.43(−0.45,17.32) | 3.62(−6.02,13.27) |
| Repeated course | 0.00(Reference) | 0.00(Reference) |
| Single-course | 18.46(−20.05,56.99) | 4.18(−31.62,39.98) |
| Time* single-course | 10.43(−0.63,21.50) | 11.15(−0.67,22.95) |
| 32weeks≤GA<37weeks^b^ | | |
| Postnatal age, week | 26.30(3.49,49.10)* | 90.23(−26.60,207.08) |
| ACS use, course |  |  |
| Non-ACS | 0.00(Reference) | 0.00(Reference) |
| Single-course | −24.25(−123.37,74.87) | −5.54(−187.51,176.43) |
| Repeated course | −61.33(−148.50,25.83) | −56.14(−206.82,94.53) |
| Time* single-course | −6.06(−43.33,31.19) | −12.05(−67.29,43.18) |
| Time* repeated course | −0.28(−32.19,31.62) | −12.15(−57.10,32.81) |
| Repeated course | 0.00(Reference) | 0.00(Reference) |
| Single-course | 37.08(−67.95,142.11) | 52.59(−122.59,227.77) |
| Time* single-course | −5.91(−44.77,32.93) | 0.17(−53.91,54.25) |

ACS, antenatal corticosteroids; GA, gestational age; IUGR, Intrauterine growth retardation.

Point estimates and 95% CI of differences in mean weight or weight growth rate are shown. *Significantly Different from reference group.

^a^ adjusted for sex, feeding pattern, cesarean section rate, time to start enteral nutrition, 5min APGAR score, GA, multiple birth, amino acid introduction, lipid introduction, duration of amino acid and lipid.

^b^ Iteration is terminated but convergence has not been achieved. Validity of the model fit is uncertain.

eTable 4 The relationship between ACS with weight changes during hospitalization in IUGR VLBW Infants

| Variable | IUGR^b^ | |
| --- | --- | --- |
|  | Unadjusted coefficient | Adjusted coefficient^a^ |
| Total | | |
| Postnatal age, week | 30.15(10.31,50.00) * | 72.02(37.40,106.64) * |
| ACS use, course |  |  |
| Non-ACS | 0.00(Reference) | 0.00(Reference) |
| Single-course | 76.04(−10.93,163.03) | 30.95(−34.70,96.61) |
| Repeated course | −61.81(−134.45,10.82) | −0.38(−57.35,56.58) |
| Time* single-course | 17.47(−9.77,44.72) | 11.96(−5.52,29.45) |
| Time* repeated course | 13.61(−9.16,36.38) | 7.93(−7.07,22.95) |
| Repeated course | 0.00(Reference) | 0.00(Reference) |
| Single-course | 138.00(44.12,231.88) * | 31.49(−42.65,105.63) |
| Time* single-course | 3.21(−16.52,22.95) | 4.03(−15.37,23.43) |
| GA<28weeks | | |
| Postnatal age, week |  |  |
| ACS use, course |  |  |
| Non-ACS | 0.00(Reference) | 0.00(Reference) |
| Single-course |  |  |
| Repeated course |  |  |
| Time* single-course |  |  |
| Time* repeated course |  |  |
| Repeated course | 0.00(Reference) | 0.00(Reference) |
| Single-course |  |  |
| Time* single-course |  |  |
| 28weeks≤GA<32weeks | | |
| Postnatal age, week | 36.28(−1120103.59,1120176.15) | 77.97(−12.37,168.31) |
| ACS use, course |  |  |
| Non-ACS | 0.00(Reference) | 0.00(Reference) |
| Single-course | −2.37(−822.58,817.83) | −30.24(−222.74,162.26) |
| Repeated course | −19.64(−426.79,387.50) | 13.14(−78.73,105.01) |
| Time* single-course | 15.91(−4099076.13,4099107.97) | 14.23(−35.66,64.14) |
| Time* repeated course | 25.12(−4110324.60,4110374.84) | 14.33(−8.89,37.55) |
| Repeated course | 0.00(Reference) | 0.00(Reference) |
| Single-course | 27.38(−205.14,259.91) | −43.38(−244.10,157.33) |
| Time* single-course | −10.31(−56.57,35.95) | −0.09(−51.99,51.80) |
| 32weeks≤GA<37weeks^b^ | | |
| Postnatal age, week | 31.44(19.06,43.82) * | 60.72(17.44,104.01) * |
| ACS use, course |  |  |
| Non-ACS | 0.00(Reference) | 0.00(Reference) |
| Single-course | 4.38(−49.47,58.24) | 23.70(−49.96,97.37) |
| Repeated course | −18.73(−72.60,35.13) | 1.65(−73.20,76.50) |
| Time* single-course | 9.51(−11.03,30.06) | 3.56(−16.62,23.76) |
| Time* repeated course | 10.11(−10.63,30.87) | 8.66(−12.01,29.34) |
| Repeated course | 0.00(Reference) | 0.00(Reference) |
| Single-course | 53.85(−28.58,136.28) | 22.23(−64.10,108.57) |
| Time* single-course | −13.63(−43.45,16.18) | −4.98(−28.95,18.97) |

ACS, antenatal corticosteroids; GA, gestational age; IUGR, Intrauterine growth retardation.

Point estimates and 95% CI of differences in mean weight or weight growth rate are shown. *Significantly Different from reference group.

^a^ adjusted for sex, feeding pattern, cesarean section rate, time to start enteral nutrition, 5min APGAR score, GA, multiple birth, amino acid introduction, lipid introduction, duration of amino acid and lipid.

^b^ Iteration is terminated but convergence has not been achieved. Validity of the model fit is uncertain. Blank cells show data which couldn’t computed.
